# P-733. Timing of Influenza and COVID-19 Vaccine Coadministration Among Adults During Two Influenza Seasons

**DOI:** 10.1093/ofid/ofae631.929

**Published:** 2025-01-29

**Authors:** Yongming Zhao, Djeneba Djibo, K J Craig, Amanda Zaleski, Dorothea Verbrugge, Cheryl McMahill-Walraven

**Affiliations:** CVS Health, Inc., Blue Bell, Pennsylvania; CVS Health, Inc., Blue Bell, Pennsylvania; fda, fdaf, Alaska; CVS, Aurora, Ohio; CVS, Aurora, Ohio; CVS Health, Inc., Blue Bell, Pennsylvania

## Abstract

**Background:**

Following the COVID-19 pandemic, little is known about timing of vaccination against both influenza (flu) and COVID-19 during peak respiratory illness season in the United States. The study objective is to examine coadministration rates of flu and COVID-19 vaccines in the two most recent flu seasons and explore sociodemographic and clinical characteristics associated with the timing.

**Table 1.**
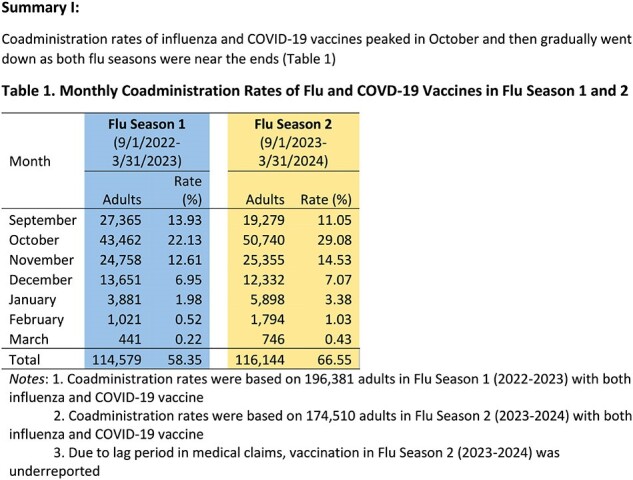
Monthly Coadministration Rates of Flu and COVD-19 Vaccines in Flu Season 1 and 2

**Methods:**

A retrospective cohort study was conducted in adults aged 18-64 years from September 2022-March 2023 or from September 2023-March 2024, enrolled in a commercial health plan with both medical and pharmacy benefit for six months before and seven months during a flu season when receiving both flu and COVID-19 vaccines. Data sources included self-reported race, enrollment records, and medical and pharmacy claims from which both vaccines were identified based on National Drug Codes and Current Procedural Terminology codes. Study subjects were categorized into two groups: coadministration with both vaccines administered on the same days or no-coadministration not on the same days. Between-group distribution analysis characterized demographics, COVID-19 diagnoses, comorbidities, and Charlson Comorbidity Index (CCI) prior to each flu season.

**Table 2.**
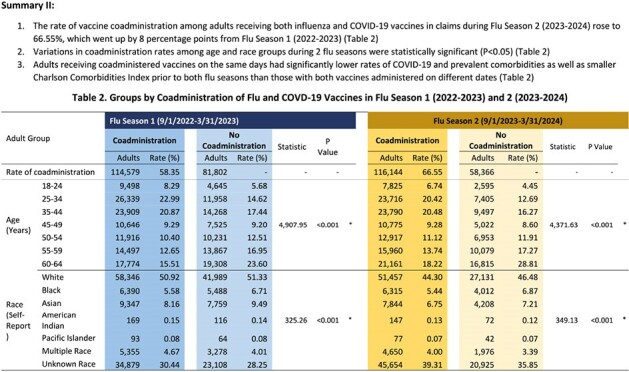
Groups by Coadministration of Flu and COVD-19 Vaccines in Flu Season 1 (2022-2023) and 2 (2023-2024)

**Results:**

In the 2022-2023 and 2023-2024 flu seasons, 196,381 and 174,510 adults received both vaccines, respectively. Coadministration rates increased from 58% (2022-2023) to 67% (2023-2024). Coadministration rates peaked in October when over 22% of adults had vaccine coadministration in both flu seasons. Variations in coadministration rates among age and race groups in both flu seasons were statistically significantly (p< 0.05). Adults with prior COVID-19 diagnoses and more comorbidities were less likely to receive co-administered vaccines in both flu seasons (p< 0.05). Adults with vaccine coadministration had lower CCI scores than those with no coadministration (p< 0.05).

**Figure:**
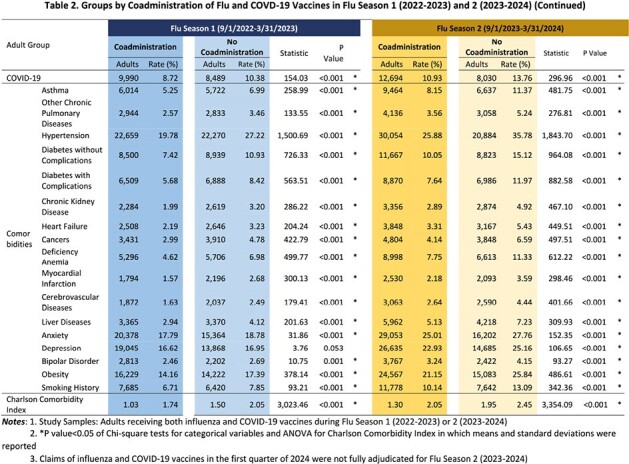

**Conclusion:**

Coadministration rate of flu and COVID-19 vaccine increases among adults in flu seasons; however, age, race, prior COVID-19 diagnoses, and more comorbidities may affect vaccine coadministration during a flu season. More research should focus on coadministration barriers in those populations.

**Disclosures:**

**Yongming Zhao, Ph.D**, CVS Health: I'm employed by CVS Health® Corporation and may own stock and/or equity|CVS Health: Stocks/Bonds (Public Company)|CVS Health: Stocks/Bonds (Public Company) **Djeneba Djibo, Ph.D**, CVS Health: I'm employed by CVS Health® Corporation and may own stock and/or equity|CVS Health: Stocks/Bonds (Public Company)|CVS Health: Stocks/Bonds (Public Company) **KJ Craig, PhD**, CVS Health: I'm employed by CVS Health® Corporation and may own stock and/or equity|CVS Health: Stocks/Bonds (Public Company)|CVS Health: Stocks/Bonds (Public Company) **Amanda Zaleski, PhD**, CVS Health: I'm employed by CVS Health® Corporation and may own stock and/or equity|CVS Health: Stocks/Bonds (Public Company)|CVS Health: Stocks/Bonds (Public Company) **Dorothea Verbrugge, MD**, CVS Health: I'm employed by CVS Health® Corporation and may own stock and/or equity|CVS Health: Stocks/Bonds (Public Company) **Cheryl McMahill-Walraven, Ph.D**, CVS Health: I'm employed by CVS Health® Corporation and may own stock and/or equity|CVS Health: Stocks/Bonds (Public Company)

